# Cannabis-Involved Traffic Injury Emergency Department Visits After Cannabis Legalization and Commercialization

**DOI:** 10.1001/jamanetworkopen.2023.31551

**Published:** 2023-09-06

**Authors:** Daniel T. Myran, Adrienne Gaudreault, Michael Pugliese, Douglas G. Manuel, Peter Tanuseputro

**Affiliations:** 1 Department of Family Medicine, University of Ottawa, Ottawa, Ontario, Canada; 2 Bruyère Research Institute, Ottawa, Ontario, Canada; 3 ICES uOttawa, Ottawa Hospital Research Institute, Ottawa, Ontario, Canada; 4 Clinical Epidemiology Program, Ottawa Hospital Research Institute, Ottawa, Ontario, Canada; 5 School of Epidemiology and Public Health, University of Ottawa, Ottawa, Ontario, Canada

## Abstract

**Importance:**

The impact of nonmedical cannabis legalization on traffic injuries and cannabis involvement in traffic injuries is unclear.

**Objective:**

To examine changes in the number and characteristics of cannabis-involved traffic injury emergency department (ED) visits from before to after legalization and subsequent commercialization (ie, increased retail store and product availability) of cannabis in Ontario, Canada.

**Design, Setting, and Participants:**

This repeated cross-sectional study examined changes in cannabis- and alcohol-involved traffic injury ED visits in Ontario, Canada, during 3 time periods: prelegalization (January 2010-September 2018), legalization with product and retail store restrictions (October 2018-February 2020), and commercialization with new products and expanded number of stores, which coincided with the COVID-19 pandemic (March 2020-December 2021). All individuals aged 16 years and older eligible for Ontario’s Universal Health Coverage were included. Season- and time-adjusted quasi-Poisson models were used to generate rate ratios with 95% CIs. Data were analyzed from March to April 2023.

**Main Outcomes and Measures:**

Quarterly counts of cannabis-involved ED visits for traffic injury.

**Results:**

There were 947 604 traffic injury ED visits, of which 426 (0.04%) had documented cannabis involvement and 7564 (0.8%) had documented alcohol involvement. Of the 418 individuals with documented cannabis involvement, 330 (78.9%) were male, 109 (25.6%) were aged 16 to 21 years (mean [SD] age at visit, 30.6 [12.0] years), and 113 (27.0%) had an ED visit or hospitalization for substance use in the 2 years before their traffic injury ED visit. Annual rates of cannabis-involved traffic injury ED visits increased 475.3% over the study period (0.18 visits per 1000 total motor vehicle collisions in 2010 to 1.01 in 2021). Over the same period, alcohol-involved traffic injury ED visits increased by 9.4% (8.03 in 2010 to 8.79 per 1000 traffic injury ED visits in 2021). Legalization with restrictions was associated with a 94% increase in the quarterly rate of cannabis involvement in traffic injury ED visits relative to prelegalization (adjusted rate ratio [aRR], 1.94; 95% CI, 1.37-2.75). Commercialization/COVID-19 was associated with a greater increase of 223% in rates (aRR, 3.23; 95% CI, 2.42-4.33). After adjusting for time trends before legalization, only commercialization/COVID-19 was associated with increased rates. Male sex (adjusted odds ratio [aOR], 3.38; 95% CI, 2.66-4.29), living in the lowest-income neighborhood (aOR, 1.92; 95% CI, 1.39-2.67), being aged 19 to 21 years (aOR, 4.67; 95% CI, 3.27-6.67), and having a prior cannabis-related ED visit (aOR, 8.03; 95% CI, 5.85-11.02) were all positively associated with cannabis involvement during a traffic injury ED visit.

**Conclusions and Relevance:**

This cross-sectional study found large increases in cannabis involvement in ED visits for traffic injury over time, which may have accelerated following nonmedical cannabis commercialization. Although the frequency of visits was rare, they may reflect broader changes in cannabis-impaired driving. Greater prevention efforts, including targeted education and policy measures, in regions with legal cannabis are indicated.

## Key Points

**Question:**

Have cannabis-involved traffic injury emergency department visits changed after cannabis legalization and the subsequent commercialization of the cannabis retail market (ie, store and product expansion) in Ontario, Canada?

**Findings:**

In this cross-sectional study capturing 426 cannabis-involved traffic injury emergency department visits, annual rates of cannabis involvement increased by 475.3% over 13 years. After accounting for time trends, legalization with restrictions was not associated with increased cannabis involvement during traffic injury emergency department visits; however, market commercialization, which overlapped with the COVID-19 pandemic, was.

**Meaning:**

These findings suggest that cannabis-involved traffic injuries have increased over time and that the commercialization of cannabis markets may result in further increases.

## Teaser

This cross-sectional study examines the number and characteristics of cannabis-involved traffic injury emergency department visits before and after cannabis legalization and commercialization in Ontario, Canada.

## Introduction

On October 17, 2018, Canada became the second country to nationally legalize recreational or nonmedical cannabis for adult use.^1^ Cannabis use, particularly intoxication, increases the immediate risk of motor vehicle collisions, and there is a major concern that legalization could increase cannabis-related impaired driving collisions.^2^ Consequently, the government of Canada set limits on blood tetrahydrocannabinol (THC) levels, with criminal charges for drivers with blood THC levels above 2 ng/mL.^3^ However, the impact of nonmedical cannabis legalization on traffic injuries in Canada and elsewhere remains unclear. In addition, to our knowledge, only a single Canadian study of 6956 injured drivers has examined a limited set of characteristics of cannabis-positive traffic injuries.^4^ More detailed data on the health characteristics of cannabis-involved traffic injuries and potential changes postlegalization are needed to target public health and clinical interventions.

Findings from the first US states to legalize cannabis have been mixed, with some studies documenting increased fatal motor vehicle collisions following legalization and others not finding any significant changes.^5,6,7,8^ Two Canadian studies have also examined population-level changes in total traffic injury visits following legalization.^9,10^ One study found no increase in traffic injury emergency department (ED) visits in Ontario and Alberta during the first year following legalization. Another study also found no increase in total traffic injury hospitalizations in Canada over 2.5 years following legalization. Critically, the slow rollout of the cannabis retail market in Canada and the overlap of the legalization period with the COVID-19 pandemic greatly reduces the ability of these studies to evaluate the impacts of legalization. During the first year and a half of cannabis legalization, the government of Canada only allowed the sale of dried cannabis flower, and there were major product and retail store shortages in Ontario (a maximum of 67 stores, 0.55 stores per 100 000 individuals aged ≥15 years, were allowed to open before April 2020).^11,12^ The legal market has since rapidly matured—with the sale of cannabis vaping products, edibles, and concentrates being permitted in January 2020 and the removal of limits on the number of cannabis stores in April 2020—with a 4.1-fold increase in per capita monthly sales ($2.67 vs $10.95 CAD per individuals aged ≥15 years) and 46.7-fold increase in per capita stores (0.20 vs 9.13 stores per 100 000 individuals aged ≥15 years) between 1 (October 2019) and 3 (October 2021) years postlegalization.^11,12,13^ However, this period of higher market maturity overlaps with the COVID-19 pandemic,^11,12,13^ during which there were large declines in motor vehicle activity, with mobility outside of the home decreasing by as much as 77% in Ontario.^14,15^ Consequently, changes in total ED visits for motor vehicle collisions during this period are likely much more influenced by enormous shifts in driving patterns during the pandemic than the changes from cannabis legalization.

This study aimed to address these gaps by examining population-level changes in the number of traffic injury ED visits with documented cannabis involvement before and after nonmedical cannabis legalization. We examined changes over distinct policy periods (prelegalization, legalization with restrictions, and legalization with commercialization/COVID-19). To adjust for overall changes in traffic mobility, we examined the proportion of total traffic injuries with documented cannabis involvement. In addition, to adjust for potential temporal changes in substance-impaired driving and COVID-19 pandemic–specific influences on substance use, we examined changes over time in traffic injury ED visits with documented alcohol involvement (control condition). Finally, we used linked individual-level data to examine a secondary objective of identifying risk factors for documented cannabis involvement during motor vehicle collisions and whether these characteristics have changed postlegalization.

## Methods

The use of the data in this cross-sectional study was authorized under section 45 of Ontario’s Personal Health Information Protection Act (PHIPA) and did not require review by a research ethics board or informed consent. This study followed the Strengthening the Reporting of Observational Studies in Epidemiology (STROBE) reporting guideline.

### Study Design, Population, and Data Sources

We used data from Ontario (Canada’s most populous province, population of 14.3 million in 2018) to conduct a repeated cross-sectional population-level study of all ED visits for traffic injuries, including motor vehicle occupants, cyclists, and pedestrians, from January 2010 to December 2021. We excluded ED visits from individuals who were younger than 16 years (minimum legal age of driving) at the time of the ED visit.

We obtained individual characteristics (age, sex, rural residence, neighborhood income quintile) and mental health and substance use (outpatient, ED, and hospital-based care) related health care in the 2 years before the traffic injury ED visit from 7 individual-level databases. The databases used in this study capture 100% of ED visits in Ontario. Databases were linked using unique coded identifiers and analyzed at ICES (formerly the Institute for Clinical Evaluative Sciences); see eAppendix in Supplement 1 for details.^16^

### Exposures

We divided our study into 3 periods: before legalization (January 2010-September 2018), after legalization but with restricted retail stores and cannabis products (hereafter, *legalization*) (October 2018-March 2020), and after legalization with unlimited retail stores and expanded products (hereafter, *commercialization*), which overlapped with the COVID-19 pandemic (April 2020-December 2021).

### Outcomes

Our primary outcome was an ED visit for a traffic injury that was positive for cannabis involvement. We first identified all ED visits for traffic injuries using *International Statistical Classification of Diseases and Related Health Problems, Tenth Revision (ICD-10)* codes for motor vehicle incidents (V20-V29, V40-V79, V30-V39, V86), and pedestrian/cyclist incidents (V01-V09, V10-V19). We then identified traffic injuries with a documented diagnosis of cannabis involvement when an *ICD-10* code for a mental and behavioral disorder due to cannabis use (F12.X) or for cannabis poisoning (T40.7) was listed as the main or contributing reason for the traffic injury ED visit. We also considered traffic injury ED visits to have cannabis involvement if a cannabis code was used during admission to the hospital or transfer to another ED. We used *International Classification of Diseases, Ninth Revision* codes (304.30 and 305.20) and the same *ICD-10 *codes for mental health hospitalizations. These cannabis codes are part of the Canadian Institute for Health Information indicator “Hospital Stays for Harm Caused by Substance Use,” which also contains further subclassification of visits (eg, acute cannabis intoxication vs cannabis-induced psychosis).^17^ We also identified traffic injury ED visits that were positive for alcohol involvement, our control condition, using *ICD-10* codes for mental and behavioral disorders due to alcohol use (F10.X) and ethanol poisoning (T51.0).^18^ As a secondary analysis, we examined motor vehicle incidents only (V20-V29, V40-V79, V30-V39, V86). Finally, we identified the clinical characteristics of traffic injury ED visits, including whether the patient arrived by ambulance and if the ED visit resulted in admission to hospital or the intensive care unit (ICU).

### Additional Covariates

At the time of each traffic injury ED visit, we identified outpatient ED visits and hospitalizations for mental health or substance use in the prior 2 years using validated diagnostic and billing codes.^18^ We also identified the neighborhood income quintile and rurality of each individual’s home address using Statistics Canada’s definitions and census data.^19,20^

### Statistical Analysis

We calculated the quarterly rate of our primary and control outcomes over the 3 policy periods (prelegalization, legalization with restrictions, and legalization with commercialization/COVID-19). We examined rates per 100 000 individuals and per 1000 traffic injury ED visits. We used adjusted quasi-Poisson models to generate rate ratios with 95% CIs comparing the restricted legalization and commercialization periods to the prelegalization rates. We generated 2 models: the first adjusted for seasonality (using an indicator for each season), and the second adjusted for seasonality and time trends with time included as a linear variable.

We captured baseline characteristics at the time of the traffic injury ED visits. For individuals with more than 1 traffic injury ED visit during the study, we captured characteristics at a randomly selected ED visit. We identified additional clinical characteristics of each traffic injury ED visit (eg, arrived by ambulance, required hospitalization). We then conducted 3 separate multivariable logistic regression analyses to identify predictors of being positive for cannabis, alcohol, or both cannabis and alcohol during a traffic injury ED visit. We included the following prespecified predictors in our model: age, rural residence, neighborhood income quintile, and past 2-year substance use and outpatient mental health visits or acute care for substance use or mental health conditions. All data were complete except for rural residence or neighborhood income quintile (<0.2% missing). We added a “missing” category for these variables and included these individuals in all analyses. All statistical analyses were completed using SAS Enterprise Guide, version 7.1 (SAS Institute).

## Results

From January 2010 to December 2021, there were 418 individuals with 1 or more cannabis-involved traffic injury ED visits in Ontario. The mean (SD) age at visit was 30.6 (12.0) years; 330 (78.9%) were male, and individuals disproportionately lived in the poorest income quintile neighborhoods (138 individuals, 33.0%). Of the 418 individuals with a cannabis-involved traffic injury ED visit, 113 (27.0%) had an ED visit or hospitalization for substance use and 84 (20.1%) for a mental health condition in the past 2 years. Compared with individuals with a traffic injury ED visit that did not involve cannabis or alcohol, individuals with a cannabis-involved ED visit were more likely to be male (78.9% vs 52.7%), younger (mean [SD] age of 30.6 [12.0] vs 41.2 [18.0]), live in lower-income neighborhoods (33.0% vs 22.2% in lowest income quintile), and have a history of substance use or mental health concerns (Table 1).

**Table 1.** Characteristics of Individuals With an ED Visit for a Traffic Injury With and Without Cannabis Alcohol Involvement From January 2010 to December 2021 in Ontario, Canada

Characteristic	No. (%)				Standardized difference (cannabis vs no alcohol or cannabis)
	Documented cannabis involvement^a^	Documented alcohol involvement^a^	Documented alcohol and cannabis involvement	No documented alcohol or cannabis involvement	
No. of individuals^b^	418	7279	177	793 469	NA
Type of trauma					
Motor vehicle collision	316 (75.6)	4591 (63.1)	135 (76.3)	597 620 (75.3)	0.01
Cyclist or pedestrian	102 (24.4)	2688 (36.9)	42 (23.7)	195 849 (24.7)	0.01
Sex					
Female	88 (21.1)	1488 (20.4)	29 (16.4)	375 639 (47.3)	0.58
Male	330 (78.9)	5791 (79.6)	148 (83.6)	417 830 (52.7)	0.58
Age, y					
16-18	45 (10.8)	258 (3.5)	12 (6.8)	58 148 (7.3)	0.12
19-21	64 (15.3)	625 (8.6)	21 (11.9)	63 058 (7.9)	0.23
22-34	182 (43.5)	2267 (31.1)	90 (50.8)	219 272 (27.6)	0.34
35-44	67 (16.0)	1268 (17.4)	28 (15.8)	127 259 (16.0)	0.00
≥45	60 (14.4)	2861 (39.3)	26 (14.7)	325 732 (41.1)	0.63
Rurality					
Urban	349 (83.5)	6012 (82.6)	153 (86.4)	689 441 (86.9)	0.10
Rural	69 (16.5)	1245 (17.1)	24 (13.6)	102 522 (12.9)	0.10
Neighborhood income quintile					
1 (Poorest)	138 (33.0)	2261 (31.1)	64 (36.2)	175 922 (22.2)	0.24
2	83 (19.9)	1471 (20.2)	36 (20.3)	164 560 (20.7)	0.02
3	77 (18.4)	1282 (17.6)	35 (19.8)	160 305 (20.2)	0.05
4	69 (16.5)	1228 (16.9)	26 (14.7)	152 897 (19.3)	0.07
5 (Richest)	49 (11.7)	963 (13.2)	<6^c^	136 690 (17.2)	0.16
Substance use acute care visit in past 2 y					
Any	113 (27.0)	1733 (23.8)	45 (25.4)	19 262 (2.4)	0.74
Alcohol	26 (6.2)	1453 (20.0)	22 (12.4)	8347 (1.1)	0.28
Opioids	10 (2.4)	72 (1.0)	<6	2196 (0.3)	0.19
Cannabis	71 (17.0)	231 (3.2)	26 (14.7)	6451 (0.8)	0.59
Other	34 (8.1)	429 (5.9)	12 (6.8)	6195 (0.8)	0.36
Mental health acute care visit in past 2 y					
Any	84 (20.1)	983 (13.5)	29 (16.4)	34 058 (4.3)	0.50
Mood disorder	37 (8.9)	348 (4.8)	12 (6.8)	11 885 (1.5)	0.34
Anxiety disorder	38 (9.1)	531 (7.3)	19 (10.7)	19 867 (2.5)	0.29
Schizophrenia/psychosis	19 (4.5)	111 (1.5)	<6	3512 (0.4)	0.27
Deliberate self-harm	17 (4.1)	306 (4.2)	<6	5620 (0.7)	0.22
Other	13 (3.1)	109 (1.5)	<6	4391 (0.6)	0.19
Outpatient mental health and addiction visit in past 2 y					
Any	225 (53.8)	3252 (44.7)	79 (44.6)	250 750 (31.6)	0.46
Family physician	210 (50.2)	3065 (42.1)	77 (42.5)	236 144 (29.8)	0.43
Psychiatrist	93 (22.2)	1055 (14.5)	28 (15.8)	59 025 (7.4)	0.43

Abbreviations: ED, emergency department; NA, not applicable.

^a^ Groups are not mutually exclusive.

^b^ Characteristics pulled at random visit for individuals with more than 1 traffic injury visit.

^c^ Results have been suppressed due to cell size to comply with privacy requirements.

Our study included 947 604 traffic injury ED visits, of which 426 (0.04%) had documented cannabis involvement. The most common cause of cannabis-involved traffic ED visits was harmful cannabis use (n = 175, 41.1%), followed by acute intoxication (n = 141, 33.1%) and cannabis dependence or withdrawal (n = 41, 9.6%). Traffic injury ED visits with cannabis involvement were more likely to arrive by ambulance (80.8% vs 41.3%) and require hospital admission (49.5% vs 6.4%) or ICU admission (21.8% vs 1.8%) compared with traffic injury ED visits without alcohol or cannabis involvement. Almost half (178, 41.8%) of cannabis-involved traffic injury ED visits also had documented alcohol involvement (Table 2). The percentage of ED visits with both cannabis and alcohol involvement arriving by ambulance or with hospital/ICU admission did not differ from cannabis involvement–only ED visits (eTable 1 in Supplement 1).

**Table 2.** Diagnostic Codes for Documented Alcohol and Cannabis Involvement During Traffic Injury ED Visits and Clinical Characteristics and Outcomes of ED Visits

	No. (%) of traffic injury ED visits		
	Documented cannabis involvement (n = 426)	Documented alcohol involvement (n = 7564)	All traffic injury ED visits (n = 947 604)
*ICD-10* diagnostic codes (code description)^a^			
F10.X or F12.X (Mental and behavioral disorders due to use of alcohol or cannabinoids)^b^	378 (88.7)	7486 (99.0)	NA
F10.0 or F12.0 (Acute intoxication)	141 (33.1)	5374 (71.0)	
F10.1 or F12.1 (Harmful use)	175 (41.1)	1265 (16.7)	
F10.2 or F12.2 (Dependence)	31 (7.3)	377 (5.0)	
F10.3, F10.4 or F12.3, F12.4 (Withdrawal)	10 (2.3)	678 (9.0)	
F10.5-F10.9 or F12.5-F12.9 (Other, unspecified and psychotic disorder)	25 (5.9)	107 (1.4)	
T51.0 or T40.7 (Poisoning by alcohol or cannabis)	20 (4.7)	51 (0.7)	
Mental health hospitalization (*ICD-9* code)	29 (6.8)	40 (0.5)	NA
Clinical characteristics and outcomes			
Arrived by ambulance	344 (80.8)	6163 (81.5)	391 655 (41.3)
Admitted to hospital	211 (49.5)	2322 (30.7)	60 480 (6.4)
Admitted to intensive care unit	93 (21.8)	952 (12.6)	17 460 (1.8)
Co-involvement of alcohol and cannabis	178 (41.8)	178 (2.4)	NA

Abbreviations: ED, emergency department; *ICD-9*, *International Classification of Diseases, Ninth Revision*; *ICD-10*, *International Statistical Classification of Diseases and Related Health Problems, Tenth Revision*; NA, not applicable.

^a^ Percentages total more than 100% because motor vehicle collisions could have more than 1 cannabis code (eg, multiple codes per visit).

^b^ F10.X = alcohol, and F12.X = cannabis.

Changes in the annual rate of cannabis- and alcohol-involved traffic injury ED visits are presented in the Figure. Over the 13-year study period, the rate of total traffic injury ED visits that involved cannabis increased by 475.3% (from 0.18 in 2010 to 1.01 per 1000 traffic injury ED visits in 2021), while the rate of total traffic injury ED visits that involved alcohol increased by 9.4% (from 8.03 in 2010 to 8.79 per 1000 traffic injury ED visits in 2021). The rate of cannabis-involved traffic injury ED visits per capita increased by 326.6% (from 0.13 in 2010 to 0.54 per 100 000 individuals in 2021), while alcohol-involved traffic injury ED visits per capita decreased by 18.9% (from 5.83 in 2010 to 4.73 per 100 000 individuals in 2021).

**Figure.**
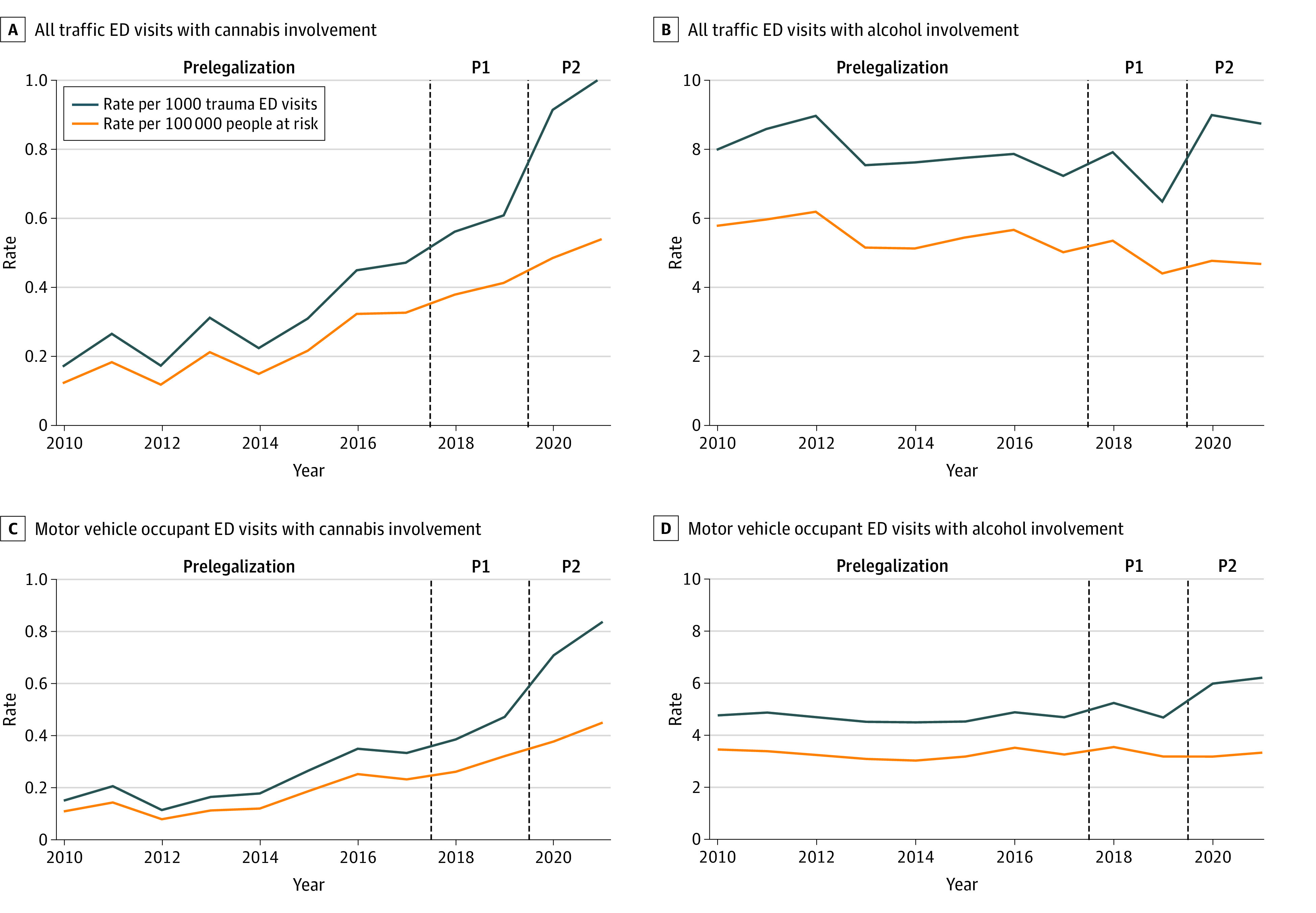
Annual Time Series Showing Rates of Traffic Injury ED Visits With Alcohol Involvement or Cannabis Involvement per 100 000 Individuals or per 1000 Traffic Injury ED Visits The dashed vertical lines divide the 3 policy periods: before legalization, after legalization with restrictions (P1), and after legalization with commercialization/COVID-19 (P2). ED indicates emergency department.

The crude mean quarterly rate of cannabis-involved traffic injury ED visits increased by 97.8% (seasonally adjusted rate ratio [a_s_RR], 1.94; 95% CI, 1.37-2.75) between the prelegalization and restricted legalization period (0.32 to 0.63 cannabis-involved ED visits per 1000 traffic injury ED visits) and by 245.0% (a_s_RR, 3.23; 95% CI, 2.42-4.33) between the prelegalization and commercialization period (0.32 to 1.09 cannabis-involved ED visits per 1000 traffic injury ED visits). In contrast, the mean quarterly rate of alcohol-involved traffic injury ED visits decreased by 13.0% (a_s_RR, 0.88; 95% CI, 0.78-0.99) between the prelegalization and restricted legalization period (7.85 to 6.83 alcohol-involved ED visits per 1000 traffic injury ED visits) and increased by 17.2% (a_s_RR, 1.12; 95% CI, 1.01-1.24) between the prelegalization and commercialization period (7.85 to 9.21 alcohol-involved ED visits per 1000 traffic injury ED visits). Increases in rates of traffic-injury ED visits with both alcohol and cannabis involvement were similar to cannabis-involved traffic injury ED visits (Table 3). Similar patterns were observed when examining cannabis- and alcohol-involved traffic injury ED visits for motor vehicle collisions only and for changes in traffic injury ED visits per 100 000 individuals (eTable 2 in Supplement 1).

**Table 3.** Changes in Overall, Cannabis-Involved, and Alcohol-Involved Traffic Injury ED Visits Before Legalization, After Legalization With Restrictions, and After Commercialization/COVID-19 in Ontario, Canada

Policy period	Before legalization (Jan 2010-Sep 2018)		Legalization with restrictions (Oct 2018-Mar 2020)		Legalization with commercialization/COVID-19 (Apr 2020-Dec 2021)		Adjusted risk ratio (95% CI)		Model adjustment
	No. of visits	Mean quarterly rate per 1000 visits	No. of visits	Mean quarterly rate per 1000 visits	No. of visits	Mean quarterly rate per 1000 visits	Legalization vs prelegalization	Commercialization vs prelegalization	
**All traffic injury ED visits**									
Any	708 821	NA	118 214	NA	120 569	NA	NA	NA	NA
Cannabis involvement	226	0.32	75	0.63	125	1.09	1.94 (1.37-2.75)	3.23 (2.42-4.33)	Season
							1.12 (0.75-1.65)	1.53 (0.99-2.36)	Season and time
Alcohol involvement	5657	7.85	807	6.83	1100	9.21	0.88 (0.78-0.99)	1.12 (1.01-1.24)	Season
							0.95 (0.82-1.09)	1.24 (1.07-1.43)	Season and time
Alcohol and cannabis involvement	92	0.02	33	0.04	53	0.06	2.01 (1.36-2.95)	2.52 (1.82-3.50)	Season
							1.47 (0.88-2.46)	1.66 (0.94-2.94)	Season and time
**Motor vehicle traffic injury ED visits**									
Any	528 810	NA	94 268	NA	81 044	NA	NA	NA	NA
Cannabis involvement	164	0.23	57	0.49	99	0.90	1.98 (1.33-2.95)	3.66 (2.63-5.11)	Season
							1.14 (0.71-1.85)	1.74 (1.03-2.96)	Season and time
Alcohol involvement	3350	4.76	584	4.98	751	6.46	1.04 (0.93-1.16)	1.33 (1.21-1.47)	Season
							1.03 (0.90-1.18)	1.32 (1.14-1.53)	Season and time
Alcohol and cannabis involvement	70	0.02	26	0.03	39	0.04	2.01 (1.25-3.22)	2.50 (1.66-3.77)	Season
							1.21 (0.65-2.25)	1.27 (0.64-2.55)	Season and time

Abbreviations: ED, emergency department; NA, not applicable.

Cannabis-involved traffic injury ED visits increased while alcohol-involved traffic injury ED visits decreased over time during the study. After adjusting for season and time trends, the increase in cannabis-involved total traffic injury ED visits was no longer significant during the restricted legalization period and was reduced in magnitude during the commercialization/COVID-19 period. The time and seasonally adjusted increase (a_st_RR) in substance-involved visits per motor vehicle traffic injuries during the commercialization/COVID-19 period remained greater for cannabis (a_st_RR, 1.74; 95% CI, 1.03-2.96) than alcohol (a_st_RR, 1.32; 95% CI, 1.14-1.53) (Table 3).

Table 4 shows the results of the univariate and multivariable logistic regression examining predictors of documented cannabis, alcohol, or cannabis and alcohol involvement in trauma ED visits. After adjustment, individuals aged 16 to 18 and 19 to 21 years, men, those living in lower-income neighborhoods, and those with prior ED visits or hospitalizations due to cannabis had the highest odds of documented cannabis involvement during traffic injury ED visits. Male sex, living in a lower-income neighborhood, and prior alcohol ED visits similarly predicted documented alcohol involvement in traffic injury ED visits, but there was no association with age.

**Table 4.** Association Between Cannabis Legalization, Demographic Factors, and Prior Health Service Use, With Alcohol and Cannabis Involvement During Traffic Injury ED Visits

	Adjusted odds ratio (95% CI)^a^		
	Documented cannabis involvement	Documented alcohol involvement	Documented alcohol and cannabis involvement
Cannabis legalization (reference = prelegalization)			
Legalization with restrictions	1.96 (1.51-2.55)	0.84 (0.78-0.91)	2.07 (1.39-3.09)
Commercialization/COVID-19	2.78 (2.22-3.47)	1.06 (0.99-1.14)	2.84 (2.02-4.01)
Type of visit (reference = pedestrian or cyclist)			
Motor vehicle collision	1.51 (1.20-1.90)	0.80 (0.76-0.84)	1.56 (1.09-2.22)
Sex (reference = female)			
Male	3.38 (2.66-4.29)	3.27 (3.08-3.47)	4.44 (2.97-6.64)
Age, y (reference = ≥45 y)			
16-18	3.76 (2.54-5.56)	0.47 (0.41-0.54)	2.44 (2.97-6.64)
19-21	4.67 (3.27-6.67)	1.05 (0.96-1.15)	3.52 (1.97-6.29)
22-34	3.65 (2.72-4.90)	1.12 (1.06-1.18)	4.22 (2.72-6.56)
35-44	2.47 (1.74-3.50)	1.08 (1.01-1.16)	2.43 (1.42-4.15)
Income quintile (reference = Q5 [richest])			
Q1 (Poorest)	1.92 (1.39-2.67)	1.53 (1.42-1.65)	2.81 (1.59-4.93)
Q2	1.32 (0.93-1.88)	1.19 (1.09-1.29)	1.82 (1.00-3.33)
Q3	1.29 (0.90-1.85)	1.12 (1.03-1.22)	1.88 (1.03-3.45)
Q4	1.21 (0.84-1.75)	1.16 (1.06-1.26)	1.49 (0.79-2.82)
Prior substance use acute care (reference = none)			
Alcohol	1.68 (1.09-2.59)	14.99 (13.97-16.08)	4.93 (2.97-8.17)
Opioids	1.54 (0.79-3.01)	0.74 (0.57-0.96)	0.72 (0.17-3.01)
Cannabis	8.03 (5.85-11.02)	1.16 (1.00-1.35)	7.34 (4.45-12.12)
Other	6.78 (0.81-56.84)	1.28 (0.29-5.57)	NA^b^
Prior mental health acute care (reference = none)			
Anxiety	0.89 (0.6-1.32)	1.25 (1.12-1.39)	1.61 (0.92-2.84)
Depression	1.61 (1.07-2.41)	1.09 (0.95-1.24)	0.84 (0.32-2.18)
Schizophrenia	1.16 (0.69-1.96)	0.72 (0.58-0.90)	1.46 (0.74-2.90)
Self-harm	1.05 (0.61-1.81)	1.76 (1.52-2.02)	0.63 (0.22-1.85)
Other	0.95 (0.51-1.75)	0.82 (0.66-1.02)	0.18 (0.02-1.36)
Prior outpatient mental health service use (reference = none)			
Family medicine	1.87 (1.50-2.31)	1.39 (1.32-1.46)	1.47 (1.05-2.05)
Psychiatry	1.46 (1.09-1.96)	1.09 (1.01-1.18)	1.00 (0.61-1.64)

Abbreviations: ED, emergency department; NA, not applicable.

^a^ Adjusted for cannabis policy period, age, sex, neighborhood income quintile, and prior outpatient or acute care for substance use and mental health.

^b^ Unstable model coefficient.

## Discussion

Over the 13-year study period, the rate of total traffic injury ED visits that involved cannabis increased by 475.3% (0.18 in 2010 to 1.01 per 1000 traffic injury ED visits in 2021). Traffic injury ED visits with documented cannabis involvement were rare and likely represent a small fraction of traffic injuries from cannabis impairment. Changes in cannabis-involved traffic injury ED visits varied based on cannabis retail policy. Increases were greater during the period that the legal market commercialized relative to the initial prelegalization period, but the overlap of the COVID-19 pandemic and the commercialization period challenges causal interpretation. Several risk factors were associated with documented cannabis involvement in traffic injury ED visits, including younger age, male sex, residing in a lower-income neighborhood, and having mental health and substance use health care visits in the past 2 years.

Our findings add to the existing literature in Canada, which to date has primarily relied on self-report or examination of overall rates of traffic injuries. Canadian surveys have not found increases in cannabis use 2 hours before driving following legalization among people who use cannabis.^21,22^ However, overall cannabis use in Ontario has increased since legalization, suggesting that the absolute number of cannabis-impaired drivers has also increased.^23^ Although cannabis-specific data are not available, a recent report found that police-reported drug-related driving collisions have increased by 109.5% since legalization (6489 in 2016-2017 to 13 595 in 2019-2020).^24^ Two studies examining total traffic injury health care visits found no change postlegalization.^9,10^ One study from British Columbia found that the prevalence of THC levels in excess of 2 ng/mL in injured drivers presenting to 4 trauma centers more than doubled following legalization (3.8% prelegalization, 8.6% postlegalization).^25^ However, since the study’s conclusion in British Columbia (the study examined changes until March 2020), the legal market has expanded considerably, with potential implications for further changes in THC detection in injured drivers.^13^ Our findings highlight that prior studies examining overall rates of traffic injuries may not have captured important impacts of cannabis legalization due to growth over time in the legal market and substantial declines in mobility during the COVID-19 pandemic.^14,15^

Our study has identified a large increase in cannabis-involved traffic injury ED visits over time. Cannabis-involved traffic injury visits were increasing prelegalization, and the period of market commercialization may have resulted in further increases in such visits. Attribution of the impacts of legalization itself from changing social norms and behaviors in the lead-up to legalization is challenging. However, the lack of increase in alcohol-involved traffic injury ED visits over the time period suggests that legalization may have played an important role in the observed increases. Our findings suggest that measures to control access to cannabis products and stores may help prevent cannabis involvement in traffic injuries. These findings are consistent with a 2021 systematic review in which 6 of 9 studies found that greater cannabis retail access was associated with increased adverse traffic-related outcomes.^26^ We highlight that the impact of cannabis legalization on road safety remains unresolved, and ongoing monitoring of various indicators concerning cannabis and injury are indicated in Canada.

Canada adopted laws aimed at preventing increases in cannabis-impaired driving following legalization.^3^ While the effectiveness of these laws remains under investigation, our study has identified several populations in which further interventions at the individual and population levels may be indicated. Consistent with prior studies,^4,27^ we found that male sex and younger age were associated with cannabis involvement during traffic injury ED visits. In Ontario, it is illegal for individuals aged 16 to 21 years to drive with detectable levels of THC or alcohol in their system. We observed that 26.1% of cannabis-involved traffic injuries compared with 12.1% of alcohol-involved traffic injuries were in individuals aged 16 to 21 years. These findings suggest that cannabis use may be particularly prevalent in young drivers and that greater education and enforcement measures may be indicated in this population. Cannabis-involved traffic injury ED visits were, on average, far more severe than injury ED visits not involving cannabis, with higher rates of hospital and ICU admission. Cannabis use may increase the risk of injury and the severity of such injuries. Alternatively, ED visits for more severe injury presentations may be more likely to have extensive investigation, including drug testing, into contributing causes. Prevention of cannabis-involved traffic injuries is a public health priority, and large increases in visits over time, regardless of the potential contribution of cannabis legalization, suggest that interventions aimed at reducing cannabis-impaired driving may be urgently indicated.

### Strengths and Limitations

Our design has several strengths. Our outcome, which captures cannabis involvement in traffic ED visits, may be less susceptible to biases than other measures used to date in Canada, such as self-reported use. While individuals may be more likely to disclose the use of cannabis following legalization, they are very unlikely to do so after a collision since cannabis use while driving continues to be illegal. Similarly, while Bill C-46 authorized police to screen for cannabis at the roadside and confirm THC levels with blood tests, this testing occurs at police stations for nonseverely injured individuals and would not influence our study outcome. Finally, access to linked individual data allowed us to identify risk factors for cannabis involvement in traffic ED visits and identify the severity of these incidents.

Nonetheless, our study has several important limitations. First, ED staff may have been more likely to test for, inquire about, and document cannabis involvement in traffic injury visits over time, particularly postlegalization. We believe these changes may explain some but not all of the observed increases for several reasons. Specifically, given the severity of these presentations, individuals likely receive extensive investigations, which would be less influenced by changes in testing practices or physician documentation over time. The findings are also consistent with large increases in cannabis use in general in the population of Ontario and with injured drivers with detectable THC levels in British Columbia.^23^ Finally, there was no immediate increase in cannabis involvement after legalization with restrictions, when there would have been increased awareness and sensitivity by ED staff for cannabis-impaired driving. Second, our outcome likely captures a small fraction of cannabis use while driving or cannabis involvement in traffic-related injuries and collisions. For example, from 2016 to 2018, there were 90 ED visits for injuries by a driver or passenger of a motor vehicle with documented cannabis involvement compared with 251 fatally injured car drivers in Ontario testing positive for cannabis.^27^ Our outcome does not capture individuals who have not used alcohol or cannabis who are injured by an alcohol- or cannabis-impaired driver, leading to further undercounting of events. Finally, our control condition is limited by uncertainty about whether alcohol and cannabis act as supplements or complements.^28^ There were also changes in the context of alcohol consumption during the pandemic (eg, closure of bars), which could have influenced alcohol-impaired driving. Regardless, our control condition does provide relevant context on shifts in alcohol-impaired driving over time.

## Conclusions

The findings of this repeated cross-sectional study suggest that cannabis-involved severe traffic injuries have increased over time. Legalization of nonmedical cannabis with widespread retail access and increased cannabis product variety may have further increased these visits despite laws specifically aimed at deterring cannabis-impaired driving. Younger adults and males appear to be at particularly increased risk of cannabis-involved traffic injuries. There is a potential need for greater interventions, including education on cannabis-impaired driving, enforcement activities, and policies to regulate access to commercial retail markets.

## Notes

Supplement 1. **eTable 1.** Clinical characteristics and outcomes of traffic injury ED visits with documented cannabis, alcohol, or both cannabis and alcohol involvement **eTable 2.** Changes in the rate of total, cannabis-, alcohol-involved, and co cannabis/alcohol-involved traffic injury ED visits per capita before-legalization, after legalization with restrictions, and after commercialization/COVID-19 in Ontario, Canada **eAppendix.** Data sources information

Supplement 2.
Data Sharing Statement
