# Supplementary material for: Cannabis-Involved Traffic Injury Emergency Department Visits After Cannabis Legalization and Commercialization
Source: JAMA Netw Open. 2023 Sep 6;6(9):e2331551. doi: 10.1001/jamanetworkopen.2023.31551 (PMC10483310; doi:10.1001/jamanetworkopen.2023.31551)
Supplement: Supplement 1. — eTable 1. Clinical characteristics and outcomes of traffic injury ED visits with documented cannabis, alcohol, or both cannabis and alcohol involvement eTable 2. Changes in the rate of total, cannabis-, alcohol-involved, and co cannabis/alcohol-involved traffic injury ED visits per capita before-legalization, after legalization with restrictions, and after commercialization/COVID-19 in Ontario, Canada eAppendix. Data sources information [file jamanetwopen-e2331551-s001.pdf]

Supplemental Online Content

Myran DT, Gaudreault A, Pugliese M, Manuel DG, Tanuseputro P. Cannabis-involved traffic injury emergency department visits after cannabis legalization and commercialization. *JAMA Netw Open.* 2023;6(8):e2331551. doi:10.1001/jamanetworkopen.2023.31551

eTable 1. Clinical characteristics and outcomes of traffic injury ED visits with documented cannabis, alcohol, or both cannabis and alcohol involvement

eTable 2. Changes in the rate of total, cannabis-, alcohol-involved, and co cannabis/alcohol-involved traffic injury ED visits per capita before-legalization, after legalization with restrictions, and after commercialization/COVID-19 in Ontario, Canada

eAppendix. Data sources information

This supplemental material has been provided by the authors to give readers additional information about their work.

© 2023 Myran DT et al. *JAMA Network Open.*

eTable 1. Clinical characteristics and outcomes of traffic injury ED visits with documented cannabis, alcohol, or both cannabis and alcohol involvement.

	Documented Cannabis Involvement (N= 426)	Documented Co-Cannabis and Alcohol Involvement (N= 178)	Documented Alcohol Involvement (N= 7,564)
	N (%) of Traffic Injury ED visits		
**Clinical Characteristics and Outcomes**			
Arrived by Ambulance	344 (80.8)	150 (84.3)	6,163 (81.5)
Admitted to Hospital	211 (49.5)	92 (51.7)	2,322 (30.7)
Admitted to Intensive Care Unit	93 (21.8)	46 (25.8)	952 (12.6)

© 2023 Myran DT et al. *JAMA Network Open.*

eTable 2. Changes in the rate of total, cannabis-, alcohol-involved, and co cannabis/alcohol-involved traffic injury ED visits per capita before-legalization, after legalization with restrictions, and after commercialization/COVID-19 in Ontario, Canada.

**Policy period**	**Before-Legalization**		**Legalization with restrictions**		**Legalization with commercialization/ COVID-19**		**Legalization vs pre-legal**	**Commercialization vs pre-legal**	
	No. Visits	Mean Quarterly Rate per 100,00 individuals	No. Visits	Mean Quarterly Rate per 100,00 individuals	No. Visits	Mean Quarterly Rate per 100,00 individuals	Adjusted Rate Ratio (95%CI)		Model Adjustment
**All Traffic Injury ED visits**									
Any	708,821	173.76	118,214	157.61	120,569	135.71	0.96 (0.91-1.01)	0.76 (0.72-0.79)	Season
							0.97 (0.91-1.03)	0.77 (0.71-0.82)	Season and Time
Cannabis Involvement	226	0.05	75	0.10	125	0.14	1.86 (1.32-2.63)	2.43 (1.82–3.24)	Season
							1.07 (0.73-1.58)	1.15 (0.75-1.76)	Season and Time
Alcohol Involvement	5,657	1.39	807	1.08	1,100	1.24	0.84 (0.76-0.94)	0.85 (0.77-0.94)	Season
							0.92 (0.81-1.04)	0.95 (0.84-1.09)	Season and Time
Alcohol /Cannabis Involvement	92	0.02	33	0.04	53	0.06	2.01 (1.36-2.95)	2.52 (1.82-3.50)	Season
							1.47 (0.88-2.46)	1.66 (0.94-2.94)	Season and Time
**Motor Vehicle Traffic ED visits**									
Any	528,810	129.63	94,268	126.71	81,044	91.17	0.99 (0.93-1.05)	0.69 (0.65-0.74)	Season
							0.98 (0.91-1.06)	0.68 (0.63-0.75)	Season and Time
Cannabis Involvement	164	0.04	57	0.08	99	0.11	1.90 (1.27-2.83)	2.71 (1.95-3.77)	Season
							1.10 (0.68-1.77)	1.30 (0.77-2.19)	Season and Time

© 2023 Myran DT et al. *JAMA Network Open.*

Alcohol Involvement	3,350	0.82	584	0.78	751	0.85	0.99 (0.90-1.10)	1.00 (0.92-1.09)	Season
							1.00 (0.88-1.13)	1.00 (0.88-1.15)	Season and Time
Alcohol/Cannabis Involvement	70	0.02	26	0.03	39	0.04	2.01 (1.25-3.22)	2.50 (1.66-3.77)	Season
							1.21 (0.65-2.25)	1.27 (0.64-2.55)	Season and Time

© 2023 Myran DT et al. *JAMA Network Open.*

eAppendix. Data Sources Information

Linked Datasets Used in Study

We obtained study data from de-identified and linked health administrative databases housed at ICES. ICES is an independent, non-profit research institute funded by an annual grant from the Ontario Ministry of Health and Long-Term Care (MOHLTC). As a prescribed entity under Ontario's privacy legislation, ICES is authorized to collect and use health care data for the purposes of health system analysis, evaluation and decision support. Secure access to these data is governed by policies and procedures that are approved by the Information and Privacy Commissioner of Ontario. In 2018, the institute formerly known as the Institute for Clinical Evaluative Sciences formally adopted the initialism ICES as its official name.

These datasets were linked using unique encoded identifiers and analyzed at ICES.

We used the following databases:

- National Ambulatory Care Reporting System (NACRS), which captures all ED visits and the cause of the visit within Ontario;
- Discharge Abstract Database (DAD), which includes records for all acute care hospitalizations in Ontario,
- Ontario Mental Health Reporting System Metadata (OMHRS) which includes all adult mental health hospitalizations in Ontario,
- OHIP Claims Database (OHIP, which captures all outpatient visits (including virtual) and the reason for visit in Ontario;,
- Registered Persons Database (RPDB), which includes the total number of persons at-risk each month and individuals' age and sex; and
- Postal Code Conversation File+ (PCCF+) which contains information on the rurality (urban vs rural) and neighborhood income for each person's home address.

© 2023 Myran DT et al. *JAMA Network Open.*
